# Exploring Intratumoral Heterogeneity in Mixed Neuroendocrine-Nonneuroendocrine Neoplasms with Spatial Transcriptomics: Even More Diverse Than Anticipated

**DOI:** 10.1007/s12022-025-09869-w

**Published:** 2025-08-20

**Authors:** Annika Weiß, Julia Teply-Szymanski, Maxime Schmitt, Sebastian Foersch, Paul Jank, Joscha Griger, Uwe Wagner, Detlef K. Bartsch, Carsten Denkert, Moritz Jesinghaus

**Affiliations:** 1 Institute of Pathology, Philipps University Marburg und University Hospital Marburg, Marburg, Germany; 2 Institute of Pathology, University Hospital Mainz, Mainz, Germany; 3 Institute of Molecular Oncology and Functional Genomics, School of Medicine, Technische Universität Muenchen, Munich, Germany; 4 Department of Gynaecology, Philipps University Marburg and University Hospital Marburg, Marburg, Germany; 5 Department of Surgery, Philipps University Marburg and University Hospital Marburg, Marburg, Germany

**Keywords:** Spatial transcriptomics, MiNEN, Mixed neuroendocrine–nonneuroendocrine neoplasms, Intratumoral heterogeneity, Gene expression, Mixed adenocarcinoma-NEC, Neuroendocrine carcinoma

## Abstract

Mixed neuroendocrine–nonneuroendocrine neoplasms (MiNEN) are usually highly aggressive tumors characterized by marked histological heterogeneity, most commonly represented by mixed adenocarcinoma and poorly differentiated neuroendocrine carcinoma (NEC). However, beyond morphological observations, the biological basis and implications of this heterogeneity remain incompletely understood. In this study, we combined component-specific next-generation sequencing and spatial transcriptomics to investigate three mixed adenocarcinoma-NEC cases from different anatomical sites (ileocecal, ovarian, gastric), tracing tumor progression from precursor lesions to invasive NEC. Genomic analyses revealed a shared trunk of driver mutations across all tumor compartments, confirming their clonal origin, while also uncovering additional compartment-specific alterations. Spatial transcriptomics, together with gene set enrichment analysis (GSEA), revealed distinct transcriptional profiles aligned with histologically annotated compartments (e.g., adenocarcinoma, NEC, precursor). In NECs, GSEA consistently showed downregulation of immune-related pathways and upregulation of proliferation-associated pathways compared to non-neuroendocrine tumor areas. Moreover, distinct transcriptomic subclusters were identified within morphologically homogeneous NEC regions in two of the three cases. These subclusters exhibited significant differences in immune regulation, proliferation signaling, and cell-cycle control, and were associated with divergent predicted chemotherapy-response signatures, suggesting clinically relevant implications for treatment sensitivity and resistance. In summary, our findings indicate that despite a shared clonal origin, MiNEN develop distinct genetic and transcriptomic features across tumor compartments. The inconsistent presence of transcriptomic subclusters within morphologically similar regions underscores the complexity of intratumoral heterogeneity in these aggressive neoplasms. By connecting morphological and molecular layers of tumor architecture, spatial profiling may aid in translating biological complexity into more targeted clinical strategies.

**Supplementary Information:**

The online version contains supplementary material available at 10.1007/s12022-025-09869-w.

## Introduction

The term tumor heterogeneity describes the biological diversity observed both within a single tumor (intratumoral) and among tumors of the same general entity (intertumoral). This diversity arises from a range of mechanisms, including genetic, epigenetic, and microenvironmental factors, and contributes to variable biological behaviors, such as differing responses to cancer therapies and variations in metastatic potential [2, 4–9, 23]. Histopathology, a key field in tumor diagnostics, vividly illustrates tumor heterogeneity, as most cancers display some degree of morphological diversity, ranging from minor cellular variations to tumors with coexisting, distinctly different components [3].

Defined by their histological heterogeneity, mixed neuroendocrine-nonneuroendocrine neoplasms (MiNEN) represent a prototypic entity for studying tumor heterogeneity from a morphological perspective. MiNEN are a conceptual category for a variety of mixed neoplasms comprising an invasive non-neuroendocrine carcinoma coexisting with a neuroendocrine neoplasm. MiNEN are usually high-grade neoplasms, most commonly represented by mixed adenocarcinoma and poorly differentiated neuroendocrine carcinoma (mixed adenocarcinoma-NEC), previously referred to as mixed adenoneuroendocrine carcinomas (MANEC). In these cases, a conventional adenocarcinoma of the respective site is combined with a poorly differentiated neuroendocrine carcinoma—either of small-cell or large-cell type—and is often accompanied by a residual precancerous precursor lesion [1, 24].

Across organ systems, mixed adenocarcinoma-NECs generally exhibit a poorer prognosis than conventional carcinomas of their respective sites and display a NEC-like biological behavior [1, 10, 13, 17, 21]. Genetic analyses have demonstrated a close relationship between mixed adenocarcinoma-NECs and conventional adenocarcinomas, with both components sharing a common mutational trunk [11, 13, 27]. Consequently, genetic mechanisms alone are unlikely to explain the dual differentiation observed in these neoplasms. Furthermore, it remains unclear whether the observed histopathological variation fully represents their underlying biological heterogeneity and whether such heterogeneity occurs not only between but also within histologically distinct tumor components.

Spatial transcriptomics, a relatively novel technology that enables the precise allocation of gene expression to distinct histological features, represents an innovative approach for investigating how morphological heterogeneity correlates with underlying biological diversity [22]. In this exploratory study, we applied spatial transcriptomics and component-specific next-generation sequencing to three MiNEN—specifically, mixed adenocarcinoma-NEC cases—from different anatomical sites (ileocecal, ovarian, gastric), tracing tumor progression from precursor lesions to invasive NEC.

Our study aimed to provide an integrated characterization of the transcriptomic landscape and evolution of MiNEN/mixed adenocarcinoma-NEC by integrating gene expression data with morphological features using spatial transcriptomics, backed by a comprehensive genetic characterization of the respective tumor components. The central hypothesis guiding our work was that the heterogeneity of MiNEN across organ systems, already substantial based on conventional histological evaluation, may extend beyond the resolution of classical morphology, with additional layers of heterogeneity emerging at the gene expression level that remain undetectable by standard histopathological methods. To assess the validity of this hypothesis, we first sought to identify transcriptomic signatures distinguishing the clearly defined histological components of these neoplasms, as well as signatures conserved across these components. Building upon these findings, we subsequently investigated whether transcriptional heterogeneity exists within histologically uniform NEC regions and examined whether morphologically similar yet transcriptionally distinct tumor areas exhibit expression patterns potentially associated with chemotherapy response.

## Patients and Methods

**Fig. 1:**
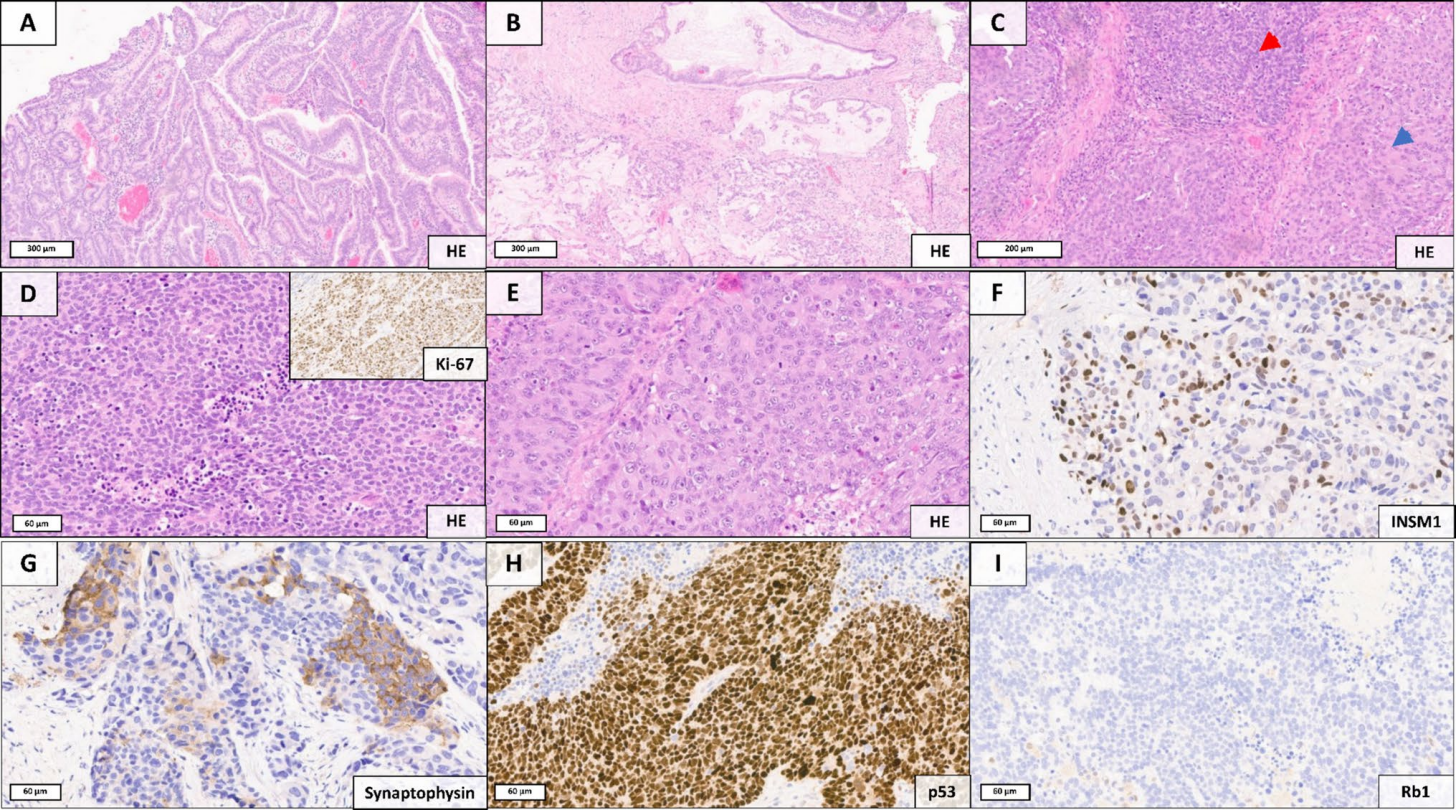
Histopathological components of the MiNEN located at the ileocecal valve, independently characterized by spatial transcriptomics and next-generation sequencing. **A** Remnants of a precursor lesion are present, represented by a tubulovillous adenoma with low-grade intraepithelial neoplasia. **B** Invasive adenocarcinoma component with partially mucinous differentiation. **C** Border region between the small-cell NEC (red arrow) and large-cell NEC (blue arrow), further illustrated in **D** (small-cell NEC, inset: Ki-67) and **E** (large-cell NEC). The NEC component shows heterogeneous expression of INSM1 (**F**), synaptophysin (**G**), and CD56 (not shown), as well as nuclear p53 overexpression (**H**) and loss of Rb1 expression (**I**)

Our exploratory approach included three patients with mixed neuroendocrine nonneuroendocrine neoplasms (MiNEN), all of which fall within the mixed adenocarcinoma-NEC category. Histological details of the respective mixed adenocarcinoma-NECs ranging from precursor lesions to the diverse invasive components, including the expression of neuroendocrine markers by the NEC components (synaptophysin, INSM1 and/or chromogranin A) are illustrated in Figs. 1 (case 1), 2 (case 2), and 3 (case 3). The gene expression (Log2FC) of neuroendocrine markers in the distinct components is listed in Supplementary Table 1.

### Case 1: Mixed Adenocarcinoma-NEC of the Ileocecal Valve

Case 1 involved a locally advanced MiNEN of the ileocecal valve in a male patient (76 years old, pT4a, pN2a). While showing a tubulovillous adenoma as its precursor lesion, the invasive neoplasm consisted of an adenocarcinoma partially exhibiting mucinous features and a neuroendocrine carcinoma (NEC) component (positive for INSM1,

CD56, and synaptophysin, negative for chromogranin A). The NEC component predominantly displayed small-cell morphology but also included areas with a large-cell phenotype (Fig. 1).

### Case 2: Mixed Adenocarcinoma-NEC of the Ovary

**Fig. 2:**
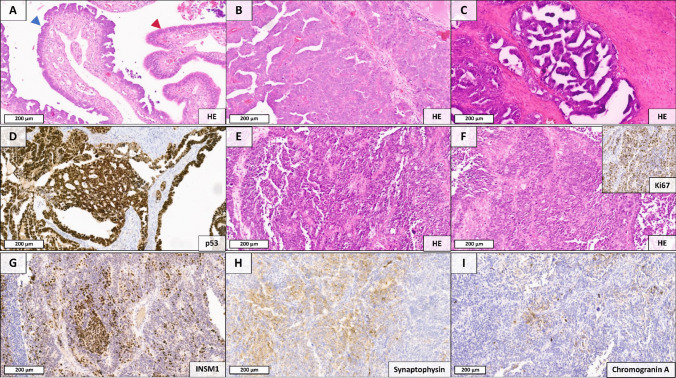
Histopathological components of the ovarian MiNEN which were characterized by spatial transcriptomics and next-generation sequencing. **A** Remnants of a serous tubal carcinoma (blue arrow) adjacent to non-neoplastic mucosa (red arrow), representing the precursor lesion of the MiNEN. **B**,** C** Invasive high-grade serous adenocarcinoma. **D** Nuclear overexpression of p53 within the adenocarcinoma component. **E**,** F** Small-cell NEC component (inset: Ki-67), showing heterogeneous but consistent expression of INSM1 **(G)** and synaptophysin **(H)**, as well as focal chromogranin A positivity **(I)**

Case 2 represented a locally advanced ovarian MiNEN (pT3c, pN1b) in a 61-year-old female patient. The tumor consisted of a high-grade serous adenocarcinoma admixed with a small cell NEC component (INSM1/synaptophysin/chromogranin A positive). In the right fallopian tube, a serous tubal intraepithelial carcinoma (STIC) was identified as the precursor lesion (Fig. 2).

### Case 3: Gastric Mixed Adenocarcinoma-NEC with Additional Sarcomatoid Component

**Fig. 3:**
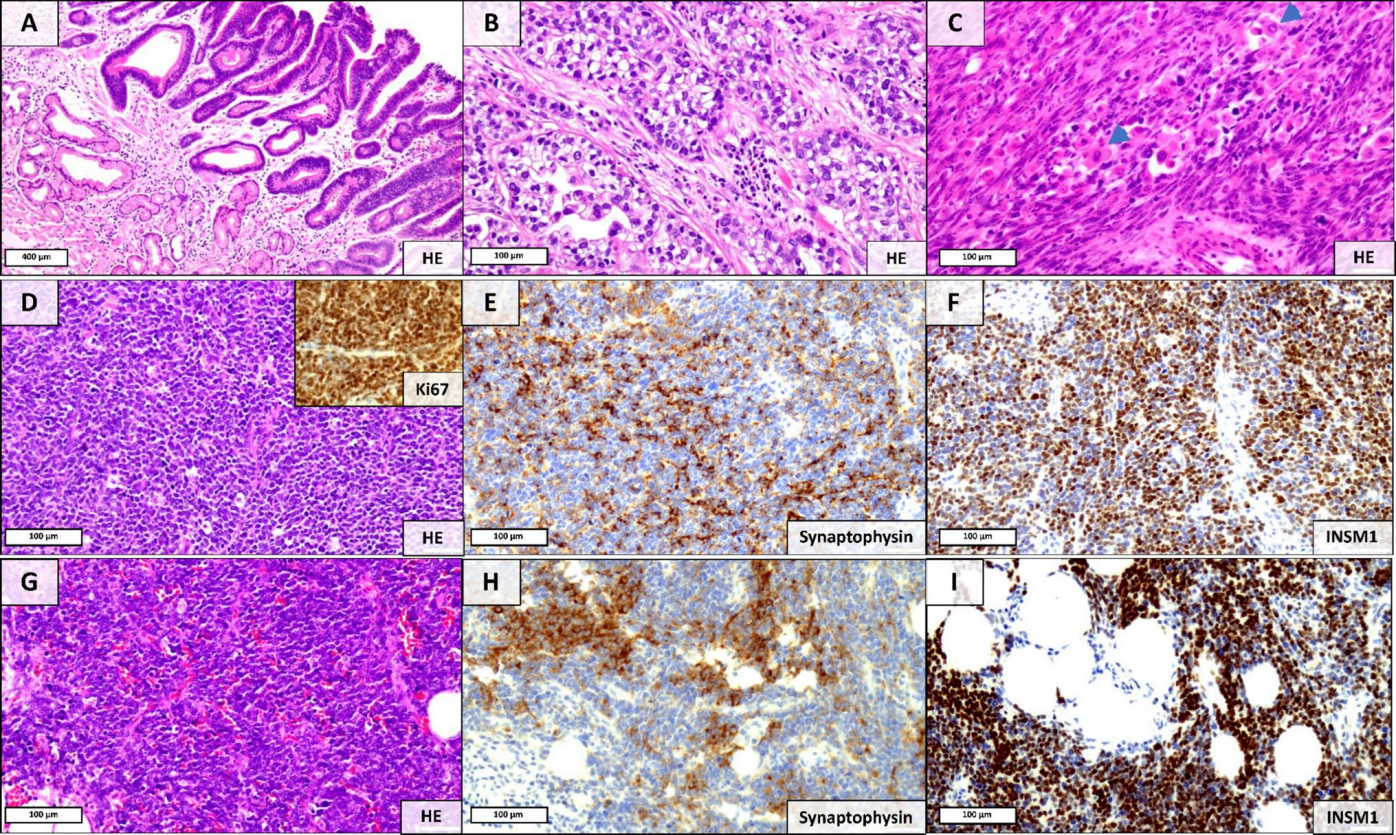
Histopathological components of the gastric MiNEN which were characterized by spatial transcriptomics and next-generation sequencing. **A** Remnants of a precursor lesion within the gastric antral mucosa, represented by a tubulovillous adenoma with low-grade intraepithelial neoplasia. **B** Invasive adenocarcinoma component with clear cell/enteroblastic morphology. **C** Spindle-cell sarcomatoid component with partial rhabdoid features (arrows)**. D**, **G** Representative areas of the small-cell NEC component (inset: Ki-67), showing immunoreactivity for synaptophysin (**E**, **H**), INSM1 (**F**,** I**), and CD56 (not shown).

Case 3 was a very large, locally advanced gastric MiNEN (pT4b, pN3b) in a female patient (75 years old). The invasive tumor consisted of an adenocarcinoma with clear cell/enteroblastic morphology [18], a small cell NEC component (INSM1/synaptophysin/CD56 positive, chromogranin A negative), and a sarcomatoid component with rhabdoid morphology. The invasive tumor was accompanied by a tubulovillous adenoma as its precursor lesion (Fig. 3).

### Next Generation Sequencing

Different tumor areas of the MiNEN were marked on H&E-stained slides by an experienced pathologist (MJ). For every MiNEN, the marked tumor areas included a precancerous lesion (case 1/3: adenoma; case 2: STIC), an adenocarcinoma component, a NEC component (separated into large and small cell in case 1), and, in case 3, an additional sarcomatoid component with rhabdoid morphology as described in detail above.

After careful microdissection of the separately marked tumor areas, genomic DNA and total RNA were semi-automatically extracted using the Maxwell RSC FFPE Plus DNA Kit and the Maxwell RSC RNA FFPE Kit, respectively, on the Maxwell RSC48 instrument (Promega).

For the detection of single nucleotide variants (SNVs), insertion-deletions (InDels), copy number variations (CNVs), and microsatellite instability (MSI), DNA libraries were prepared with the VariantPlex® (VP) Pan Solid Tumor panel (VP-PST, 185 genes, ArcherDX/Integrated DNA Technologies [IDT]) following the manufacturer’s protocol.

Correspondingly, RNA libraries were prepared from the same regions using the FusionPlex® Pan Solid Tumor panel (FP-PST, 137 genes, ArcherDX/IDT) to identify fusions and exon-skipping events. Libraries were quantified with the NEBNext Library Quant Kit for Illumina (New England Biolabs), pooled, and paired-end sequenced for 151 cycles on a NextSeq550 DX or NovaSeq6000 sequencer (Illumina, Inc.). Secondary analysis was performed on the Archer Analysis platform, and variants were called if they passed the following filters: read depth ≥ 100, variant allele frequency (VAF) ≥ 5%, and gnomAD global population frequency ≤ 1%. For variant classification, the tertiary analysis software Molecular Health Guide (MHG, Molecular Health) was used.

### Spatial Gene Expression Profiling of Whole Slides

Spatial transcriptomics analysis was conducted using the Visium Spatial Gene Expression for FFPE assay (10 × Genomics). RNA integrity was verified to meet the quality threshold (DV200 > 50%). FFPE tissue blocks were scored to fit the 6.5 mm × 6.5 mm capture areas and sectioned into 5 µm slices. Three capture areas were used for both the ileum and ovarian cancer cases, while six capture areas were employed for the gastric cancer case. Following deparaffinization, H&E staining, and imaging, library construction was executed according to manufacturer specifications. Subsequently, libraries underwent sequencing on the Illumina NextSeq550 platform to achieve a minimum depth of 25,000 mean read pairs per spot. Sequencing data were processed using the SpaceRanger pipeline (V.2.0.1.) and aligned to the GRChr38 transcriptome. Manual fiducial alignment and pathologist annotations for downstream analyses were facilitated using Loupe Browser v7.0.1. Only tumor regions were annotated and included in further analyses, resulting in 6183 spots for the ileum, 3779 for the ovary, and 16,578 for the stomach.

### Differential Gene Expression Analysis and Gene Set Enrichment Analysis

All analyses were conducted using R (v.4.4.1). Spatial transcriptomic data were preprocessed by filtering out spots with fewer than 200 detected genes to ensure data quality. The Seurat package (v.5.1.0) was used to identify differentially expressed genes across clusters and annotated subtypes, applying the FindAllMarkers() function with default settings. The semla package (v.1.2.1) was utilized for spatial transcriptomics analysis and visualization. Gene set enrichment analysis (GSEA) was performed using the fgsea package (version 1.30.0), with MSigDB utilized for the Hallmark analysis. For the NEC therapy analysis, drug signatures were extracted from the DSigDB database (https://dsigdb.tanlab.org/DSigDBv1.0/) [29], specifically focusing on compounds relevant to NEC treatment, which were then used as reference datasets for GSEA.

## Results

### Case 1: Mixed Adenocarcinoma-NEC of the Ileocecal Valve

#### Genetic Profile of the Different Tumor Components

**Fig. 4:**
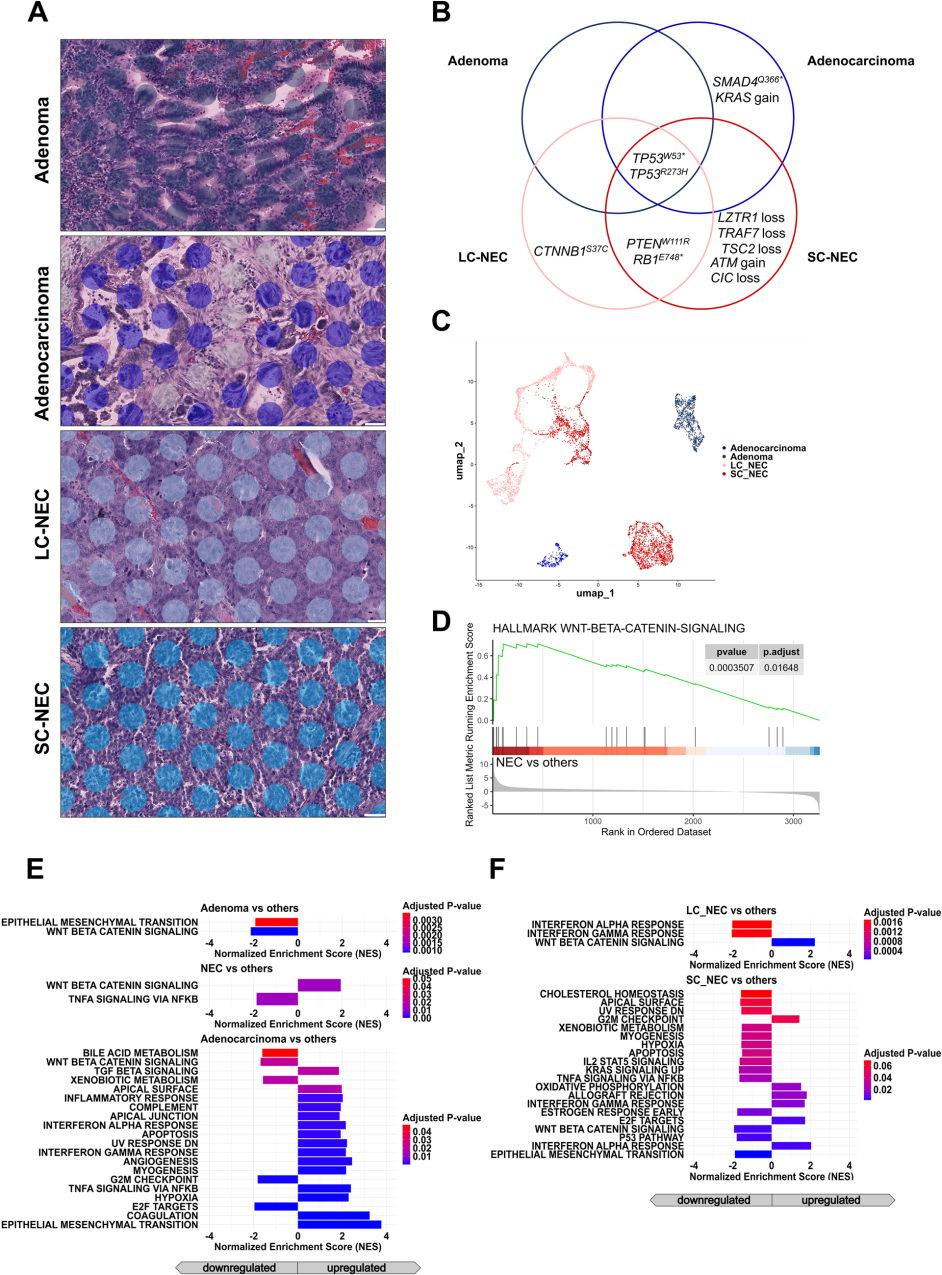
Analysis of intratumoral heterogeneity in mixed adenocarcinoma-NEC of the ileocecal valve. **A** Representative H&E-stained sections of the four distinct histologic components identified within a single tumor: adenoma, adenocarcinoma, large cell neuroendocrine carcinoma (LC-NEC), and small cell neuroendocrine carcinoma (SC-NEC). Visium spatial transcriptomic spots are overlaid. (Scale bar: 50 µm). **B** Venn diagram summarizing somatic mutations and copy number alterations detected in each component. Shared *TP53* mutations suggest a common clonal origin, while component-specific alterations reflect divergent molecular evolution and lineage commitment. **C** UMAP projection of transcriptomic profiles from spatially resolved spots demonstrates distinct clustering by histologic subtype, indicating transcriptional divergence across tumor components. **D** Enrichment plot demonstrating the key gene set activation, reflecting subtype-specific pathway dynamics. **E**, **F** Bar plots depict the normalized enrichment scores for all significantly upregulated and downregulated gene sets (*p.* adj. < 0.05), highlighting differences in pathway activities across subtypes. NEC components demonstrate significant enrichment of Wnt/β‑catenin signaling, whereas adenocarcinomas are characterized by increased inflammatory and proliferative signaling (**E**). Subdivision of the NEC compartment into large‑cell NEC (LC‑NEC) and small‑cell NEC (SC‑NEC) shows that Wnt/β‑catenin pathway enrichment is concentrated in LC‑NEC, while SC‑NEC exhibits upregulation of immune‑response–related pathways (**F**).

As depicted in Fig. 4B, next-generation sequencing of all tumor parts revealed a shared mutational trunk characterized by two *TP53* mutations present throughout. The NEC component exhibited additional inactivating variants in *PTEN* and *RB1*, alongside an exclusive *CTNNB1* mutation in the LC-NEC component. Meanwhile, the SC-NEC compartment harbored several exclusive copy number variations. The adenocarcinoma component showed an exclusive amplification of *KRAS* as well as a *SMAD4* mutation.

#### Spatial Transcriptomics of the General Tumor Components

Spatial transcriptomic analysis of the annotated spots revealed distinct clusters corresponding to the different tumor compartments, aligning with their respective histological characteristics (Fig. 4C). Gene Set Enrichment Analysis (GSEA) demonstrated significant transcriptomic differences across the various tumor regions. Figure 4E highlights key gene sets identified through GSEA: The adenocarcinoma was marked by a pronounced immune-associated (interferon α response, interferon γ response, inflammatory response) and epithelial-mesenchymal transition (EMT) signature.

The SC-NEC exhibited a prominent proliferation signature, characterized by downregulated apoptotic and p53 pathways alongside upregulated cell-cycle activity, including E2F and G2M signaling. In contrast, activated Wnt signaling was observed in the LC-NEC.

#### Transcriptomic Subclusters Within the LC-NEC Component

**Fig. 5:**
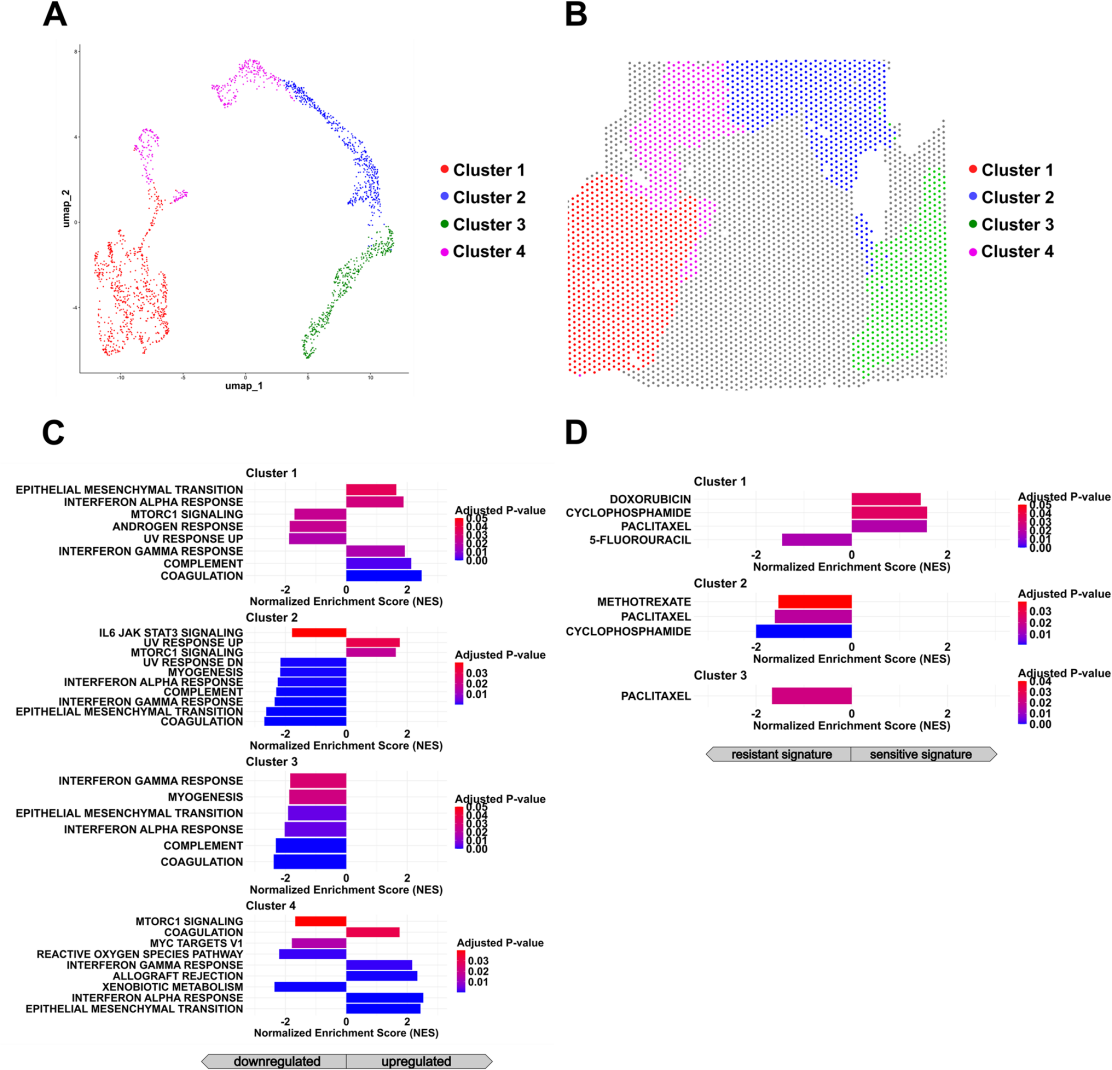
Identification of transcriptomic subclusters in the LC-NEC compartment of mixed adenocarcinoma-NEC of the ileocecal valve. **A** UMAP projection of spatial transcriptomic data from the LC-NEC region reveals four transcriptionally distinct subclusters identified through unsupervised clustering. **B** Spatial mapping of LC-NEC subclusters demonstrates regionally segregated distribution patterns within the tumor. **C**,** D** Gene set enrichment analysis (GSEA) indicates distinct cluster-specific signatures. Bar plots present the normalized enrichment scores for all significantly upregulated and downregulated gene sets (*p*. adj. < 0.05). **C** GSEA utilizing Hallmark gene sets reveals that Cluster 1 and 4 are enriched for epithelial-mesenchymal transition (EMT) and immune signaling pathways, while Cluster 2 is marked by UV response and mTORC1 signaling. **D** GSEA employing drug response signatures suggests potential differences in therapeutic sensitivity among LC-NEC subclusters.

By utilizing spatial transcriptomics and unsupervised clustering on regions identified as LC-NEC, we identified four distinct subclusters (Fig. 5A, B). Gene set enrichment analysis (GSEA) of the differentially expressed genes revealed that Clusters 1 and 4 were enriched in immune response-related gene sets, including those associated with interferon α and γ responses, as well as the complement Hallmark gene set. In contrast, Cluster 2 was primarily characterized by a cellular stress response, encompassing UV response and mTORC1 signaling (Fig. 5C). When examining drug signatures representing current treatment options, the LC-NEC subclusters exhibited unique profiles, for example, suggesting that Cluster 1 may show greater sensitivity to paclitaxel compared to Clusters 2 and 3 (Fig. 5D).

#### Transcriptomic Subclusters Within the SC-NEC Component

**Fig. 6:**
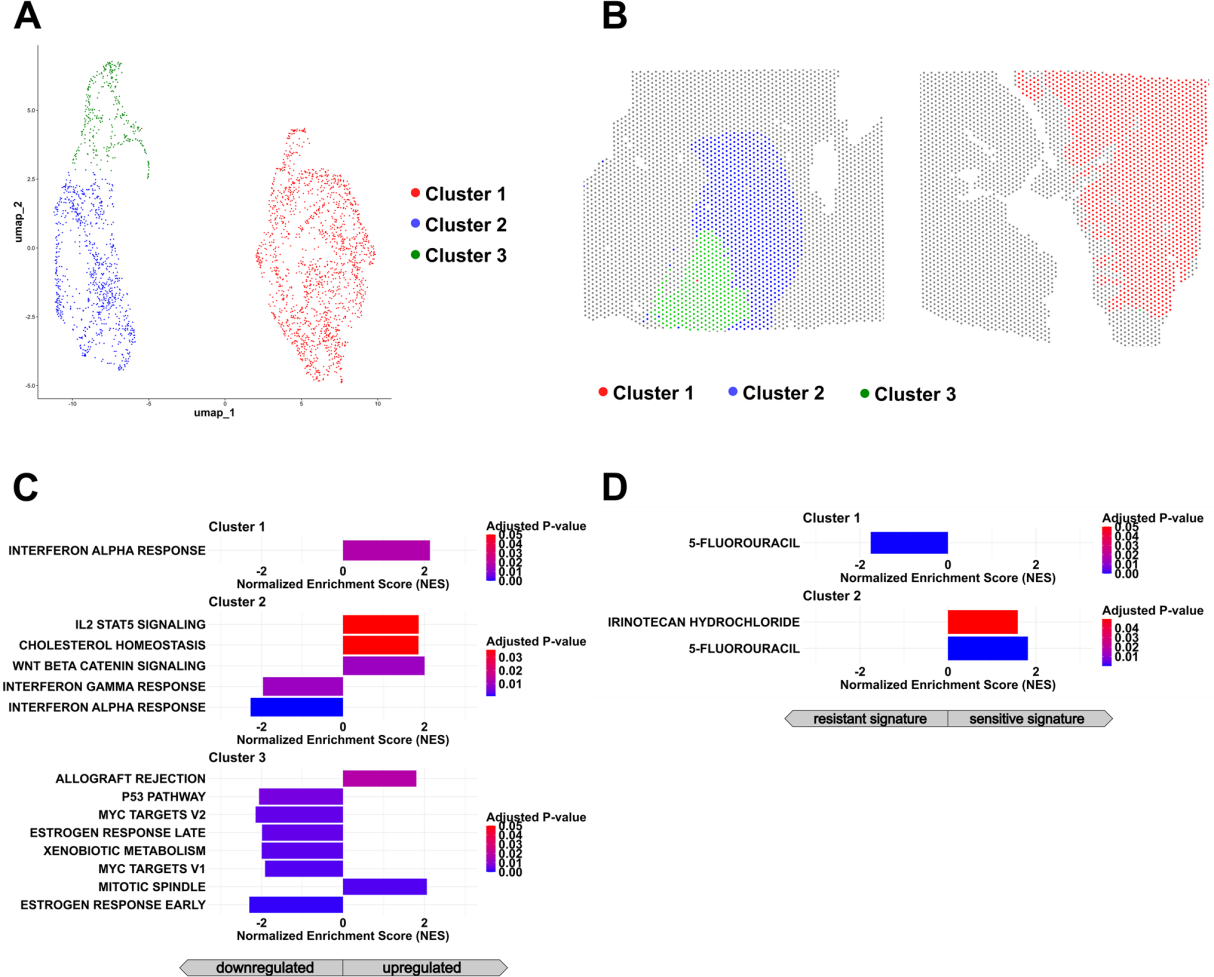
Identification of SC-NEC subclusters in the neuroendocrine compartment mixed adenocarcinoma-NEC of the ileocecal valve. **A** UMAP projection of spatial transcriptomic profiles from the SC-NEC region identifies three transcriptionally distinct subclusters through unsupervised clustering. **B** Spatial localization of SC-NEC subclusters shows defined distribution patterns, suggesting regional heterogeneity within the small cell neuroendocrine compartment. **C**, **D** Gene set enrichment analysis reveals cluster-specific signatures. Bar plots display the normalized enrichment scores for all significantly upregulated and downregulated gene sets (*p*. adj. < 0.05). **C** Hallmark GSEA demonstrates that Cluster 1 is enriched for interferon α signaling, Cluster 2 shows activation of IL2-STAT5 and Wnt/β-catenin pathways, and Cluster 3 exhibits downregulation of p53 signaling, Myc targets, and estrogen response pathways. **D** Drug signature enrichment analysis suggests potential transcriptional differences in sensitivity to selected therapeutic agents, including 5-fluorouracil and irinotecan.

In the SC-NEC compartment, unsupervised clustering resulted in the identification of three distinct subclusters (Fig. 6A, B). Similar to the large cell counterpart, the SC-NEC subclusters exhibited unique enrichment profiles for certain Hallmark gene sets. Clusters 1 and 3 displayed stronger immune-responsive profiles, with enrichment in the interferon α response and allograft rejection pathways, respectively. In contrast, Cluster 2 was characterized by enrichment in interleukin-2, STAT5 signaling, and Wnt/β-catenin signaling (Fig. 6D). Additionally, analysis of drug signature datasets suggested that regions belonging to Cluster 2 may demonstrate greater sensitivity to treatment with 5-fluorouracil and irinotecan compared to the other SC-NEC regions (Fig. 6D).

### Case 2: Mixed Adenocarcinoma-NEC of the Ovary

#### Genetic Profile of the Different Tumor Components

**Fig. 7:**
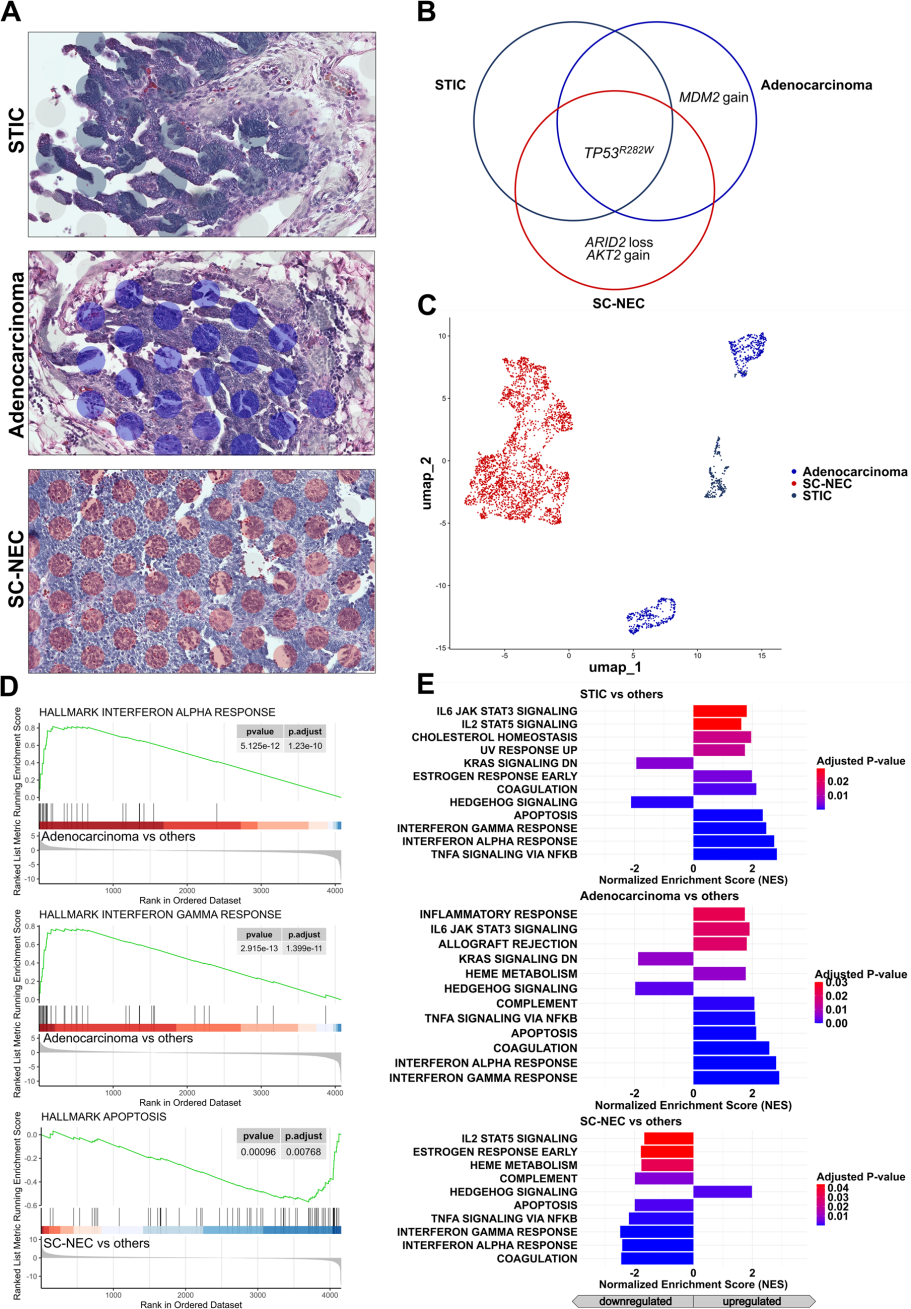
Analysis of intratumoral heterogeneity in mixed adenocarcinoma-NEC of the ovary. **A** Representative histologic components including Serous tubal intraepithelial carcinoma (STIC), high-grade serous adenocarcinoma and small cell neuroendocrine carcinoma (SC-NEC) with Visium spatial transcriptomic spots overlaid (Scale bar: 50 µm). **B **Somatic mutations and copy number alterations identified in each tumor compartment reveal both overlapping and distinct genomic alterations.** C **UMAP projection of spatial transcriptomic data shows transcriptional segregation of the tumor components.** D, E **Gene Set Enrichment Analyses reveal distinct region-specific signaling programs within the tumor.** D **Enrichment plots highlight key signaling pathways differentially regulated in the respective components.** E **Bar plots display the Normalized Enrichment Scores for significantly upregulated pathways (*p.* adj. <0.05), underscoring the differential signaling dynamics across regions. STIC and adenocarcinoma regions display upregulated immune response pathways, whereas the SC-NEC compartment is characterized by enhanced hedgehog signaling and attenuated apoptotic features.

The different tumor parts revealed a shared *TP53* mutation across all components. An exclusive *MDM2* amplification was identified within the serous adenocarcinoma fraction, while an *ARID2* loss and an *AKT2* amplification were uniquely present in the SC-NEC compartment (Fig. 7B).

#### Spatial Transcriptomics of the General Tumor Components

Spatial transcriptomic analysis of the annotated spots revealed distinct clusters corresponding to the different tumor compartments, aligning with their respective histological characteristics (Fig. 7C). Notably, one adenocarcinoma cluster was defined by an extensive MDM2 signaling, restricted to this cluster alone.

GSEA identified downregulation of apoptotic pathways as the transcriptomic hallmark of SC-NEC (Fig. 7D). In contrast, a pronounced immune response signature was detected in both STIC and the adenocarcinoma, marked by an interferon α and interferon γ response in the adenocarcinoma (Fig. 7D). Further, GSEA identified complementary pathway alterations: SC-NEC displayed downregulated immune pathways alongside activated Hedgehog signaling, while STIC and the adenocarcinoma showed additional upregulation of immune-related pathways (Fig. 7E).

#### Transcriptomic Subclusters Within the SC-NEC Component

**Fig. 8:**
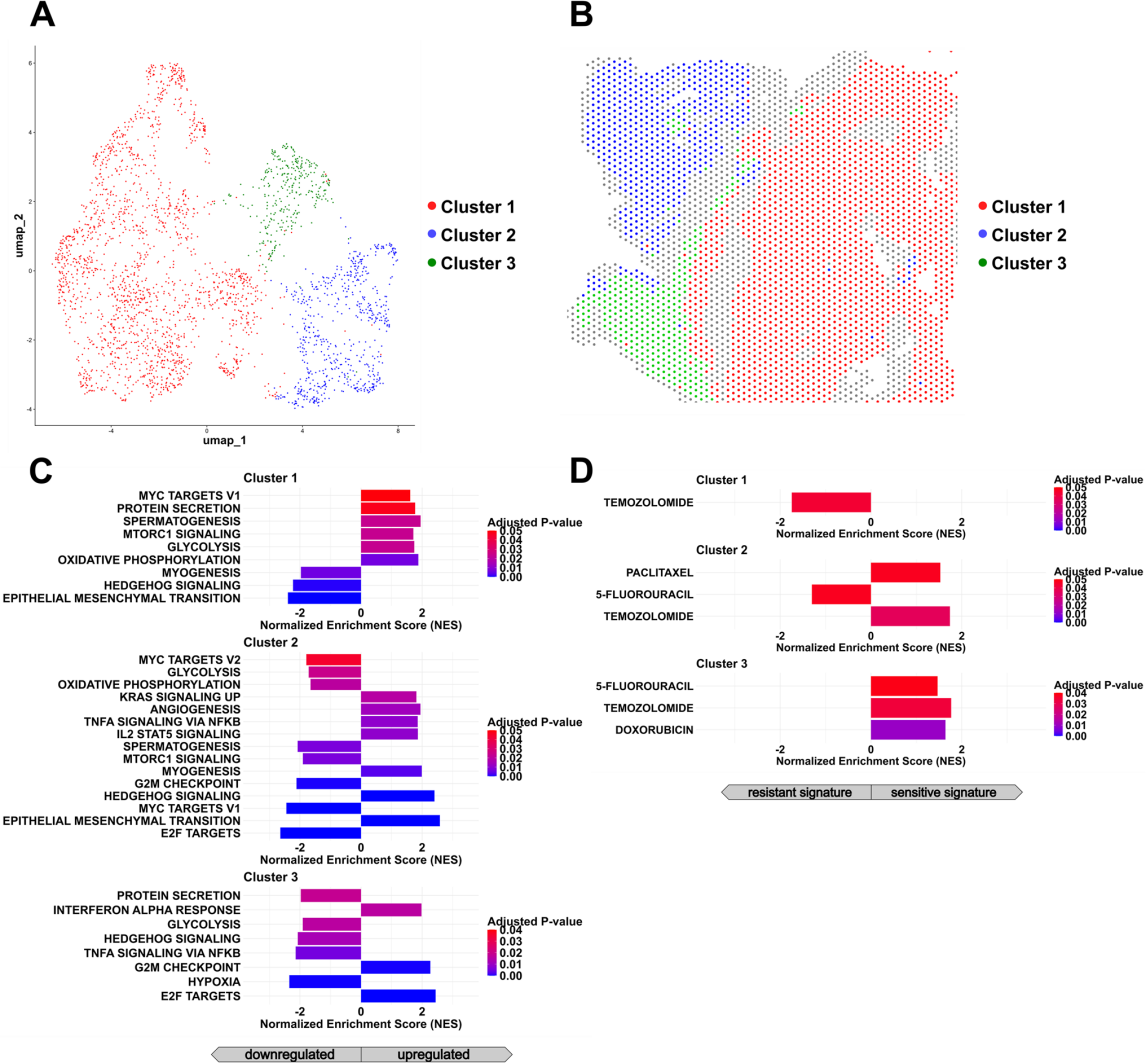
Identification of subclusters in the neuroendocrine compartment of mixed adenocarcinoma-NEC of the ovary. **A** UMAP projection of spatial transcriptomic data from the SC-NEC region identifies three transcriptionally distinct subclusters via unsupervised clustering. **B** Spatial localization of SC-NEC subclusters reveals regionally segregated distribution patterns within the tumor. **C**, **D** Bar plots display the normalized enrichment scores for significantly upregulated pathways across subclusters (*p*. adj. < 0.05). **C** Gene set enrichment analysis (GSEA) of Hallmark pathways highlights subcluster-specific biological programs, with Cluster 1 enriched for metabolic pathways, Cluster 2 exhibiting increased signaling activity and myogenesis-related signatures, and Cluster 3 characterized by enrichment of interferon α response and cell cycle regulatory pathways. **D** Drug signature enrichment analysis reveals potential subcluster-specific differences in therapeutic sensitivity within the SC-NEC compartment.

We identified three distinct transcriptomic clusters within the SC-NEC component (Fig. 8A), with their spatial distribution shown in Fig. 8B. While these clusters shared the general features of the broader SC-NEC profile in comparison to other tumor components, notable differences emerged between them. Cluster 1 was characterized by proliferation and metabolic pathways, including Myc activation.

Cluster 2 exhibited an accentuated cell signaling signature alongside exclusive upregulation of the Hedgehog pathway. In contrast, Cluster 3 displayed an activated cell-cycle signature marked by E2F and G2M signaling (Fig. 8C).

Integration of the transcriptomic profiles with a drug signature database revealed distinct patterns of predicted treatment response. While sensitivity signatures for temozolomide, doxorubicin, and 5-fluorouracil were distributed across multiple clusters, only Cluster 2 showed signs of potential sensitivity to paclitaxel (Fig. 8D), which was administered to the patient.

### Case 3: Gastric Mixed Adenocarcinoma-NEC with Additional Sarcomatoid Component

#### Genetic Profile of the Different Tumor Components

**Fig. 9:**
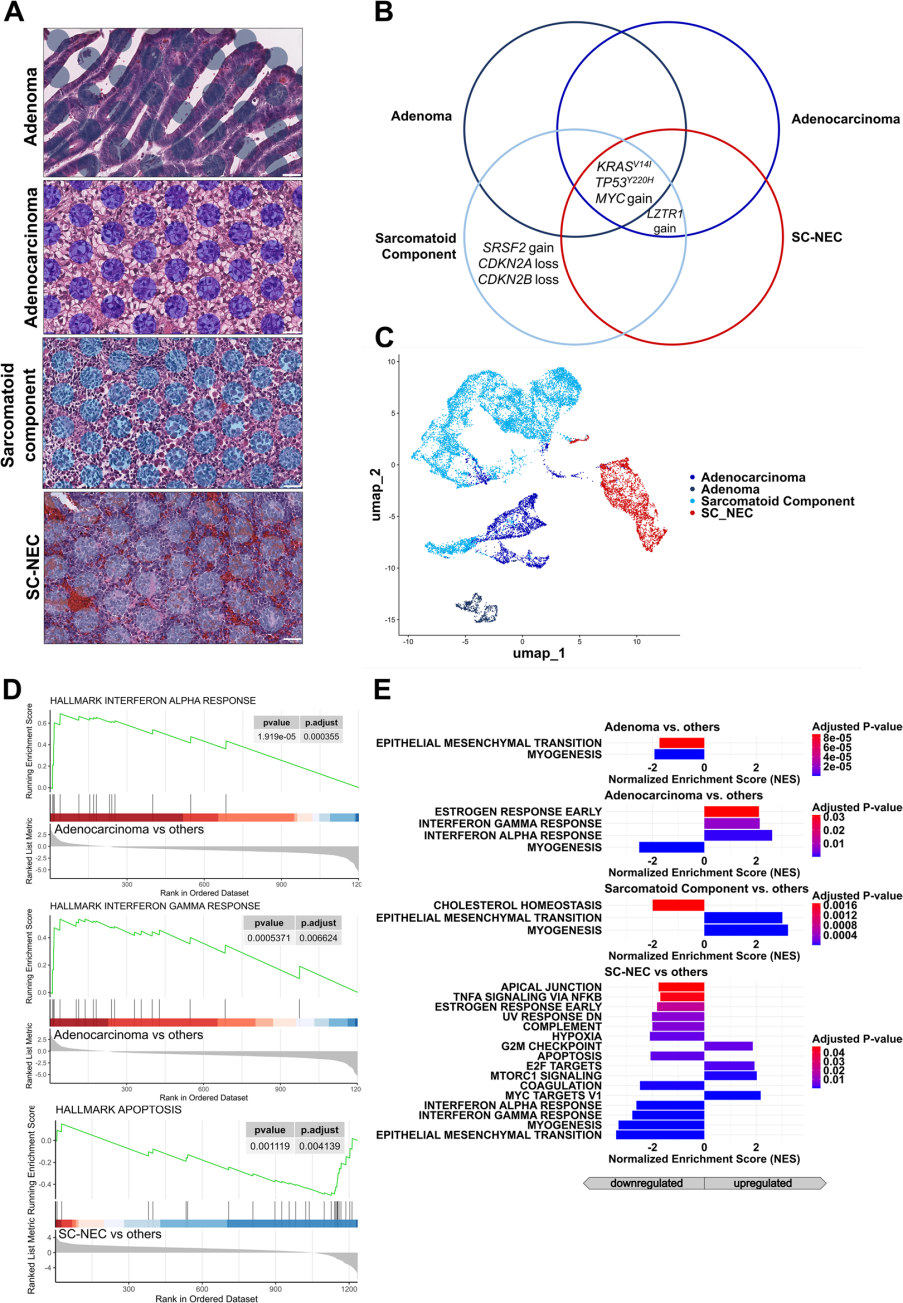
Analysis of intratumoral heterogeneity in gastric mixed adenocarcinoma-NEC. **A **Representative histologic sections illustrating the four distinct tumor components: adenoma, adenocarcinoma, small cell neuroendocrine carcinoma (SC-NEC), and a sarcomatoid component. Visium spatial transcriptomic spots are overlaid. (Scale bar: 50 µm). **B **Venn diagram of somatic mutations and copy number alterations identified in each component. All regions share *TP53* and *KRAS* mutations as well as *MYC* copy number gain, suggesting a common clonal origin. **C **UMAP projection of spatial transcriptomic data reveals transcriptionally distinct clusters corresponding to histologic subtypes.** D**,** E **Gene set enrichment analysis highlights component-specific pathway activation.** D **Enrichment plots display key gene sets up-/downregulated in the respective components.** E **Bar showing the Normalized Enrichment Scores (NES) indicate increased activation in SC-NEC of Myc targets, G2M checkpoint, E2F, and mTORC1 signaling pathways.

Sequencing across all compartments, ranging from the premalignant adenoma to the sarcomatoid component, revealed shared *TP53* and *KRAS* mutations as well as a *MYC* amplification. All invasive regions exhibited an *LZTR1* amplification. Notably, the copy number gains of both *MYC* and *LZTR1* were particularly pronounced in the SC-NEC (*MYC*: 25.0, *LZTR1*: 56.16) and sarcomatoid areas (*MYC*: 29.02, *LZTR1*: 58.18) compared to the adenocarcinoma (*MYC*: 14.3, *LZTR1*: 22.88) and adenoma (*MYC*: 10.13, *LZTR1*: none) compartment (Fig. 9B).

#### Spatial Transcriptomics of the General Tumor Components

Spatial transcriptomic analysis of the annotated tumor spots demonstrated distinct transcriptomic profiles defining the tumor compartments (Fig. 9C). Gene set enrichment analysis (GSEA) using Hallmark gene sets indicated significant enrichment of immune response-related gene sets, such as the interferon α and γ responses, within the adenocarcinoma compartment (Fig. 9D, E). In contrast, the sarcomatoid component exhibited strong enrichment in myogenesis-associated genes (Fig. 9E). The SC-NEC component showed pronounced enrichment in gene sets related to cell cycle regulation, including the G2M checkpoint and E2F targets. Notably, despite the presence of *MYC* amplification across all tumor compartments (Fig. 9B), the SC-NEC area of the gastric MiNEN displayed particularly strong enrichment of Myc targets compared to the other tumor regions (Fig. 9E).

#### Transcriptomic Subclusters Within the NEC Component

**Fig. 10:**
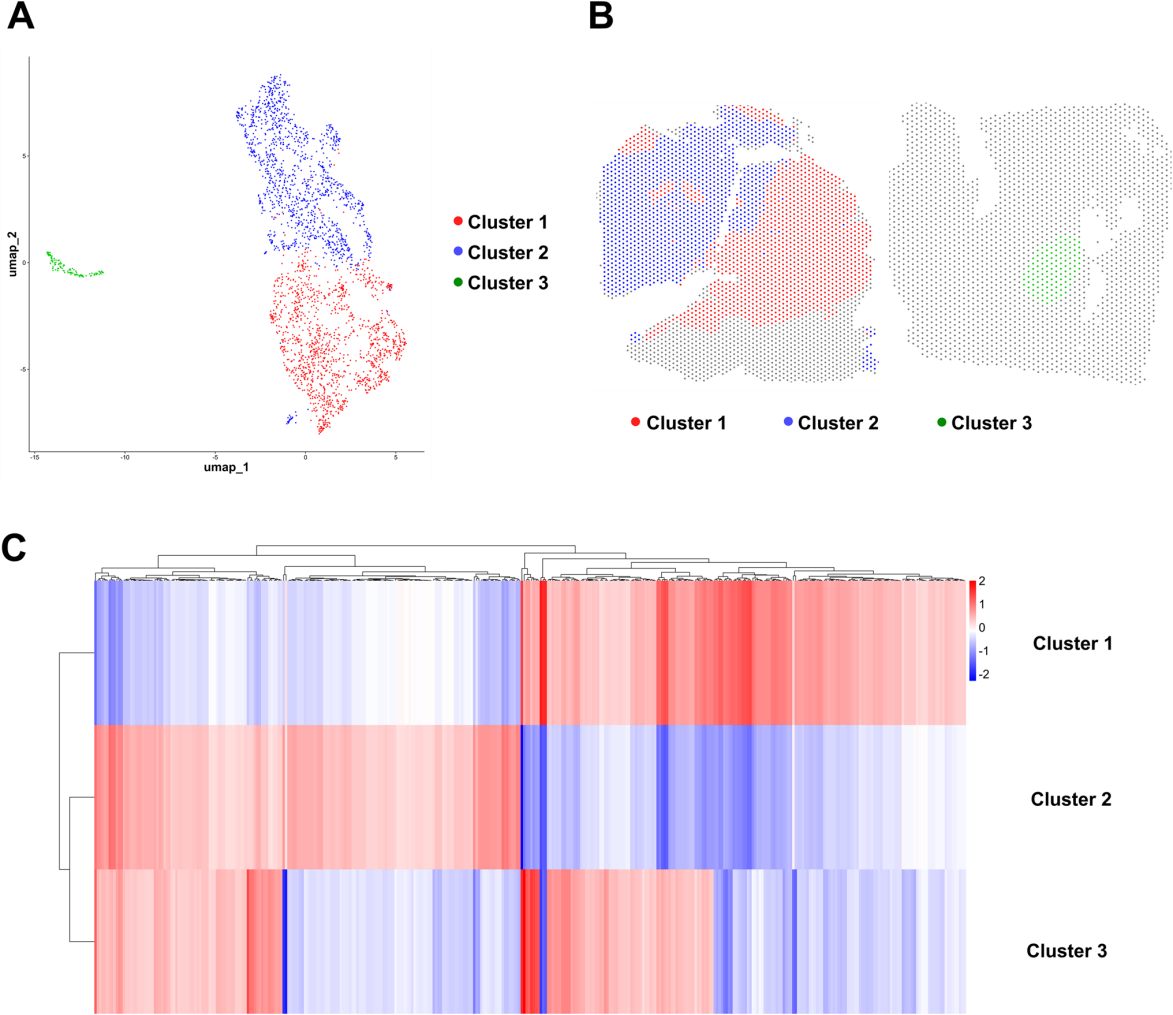
Identification of subclusters in the neuroendocrine compartment of gastric mixed adenocarcinoma-NEC. **A **Unsupervised clustering of spatial transcriptomic data from the neuroendocrine (NEC) region reveals distinct transcriptional subclusters.** B **Spatial mapping of NEC subclusters demonstrates regionally distinct localization patterns within the tumor.** C **Heatmap illustrating the top 500 differentially expressed genes (adjusted p < 0.05) across NEC subclusters, highlighting transcriptional heterogeneity within the neuroendocrine compartment. However, in contrast to the other MiNEN cases, these genes showed a heterogeneous distribution and did not cluster within any defined gene set

Unsupervised clustering of the SC-NEC compartment identified three distinct subclusters (Fig. 10A, B). These subclusters exhibited differentially expressed genes, with the top 500 genes per cluster presented in the heatmap (Fig. 10C). However, in contrast to the previously discussed cases in the ovary and ileum, there was no significant enrichment observed for either Hallmark gene sets or drug signatures.

## Discussion

Mixed neuroendocrine nonneuroendocrine neoplasms (MiNEN) represent an ideal model for studying intratumoral heterogeneity from a histological perspective, given their defining feature—the coexistence of distinct non-neuroendocrine carcinoma and NEC regions within the same tumor. Despite recognition of their morphological complexity, the true extent and biological implications of this heterogeneity remain incompletely understood [1, 11, 12, 15–17, 20, 27].

The starting point of this study was our hypothesis that the heterogeneity of MiNEN across organ systems, already substantial based on conventional histological assessment, may extend beyond what is detectable by standard morphology, with additional layers of heterogeneity emerging at the level of gene expression. Therefore, we combined deep next-generation sequencing (NGS) and spatial transcriptomics (ST) to investigate the molecular evolution of three MiNEN cases, specifically mixed adenocarcinoma-NEC, tracing tumor progression from precursor lesions to NEC. The rationale for selecting MiNEN of different origins was to test our main hypothesis not only within a single anatomical location, but to obtain a broader perspective on the heterogeneity of MiNEN as a tumor category. Our integrative approach revealed previously unrecognized complexity, demonstrating that intratumoral heterogeneity in these neoplasms exceeds prior assumptions.

Genomic analyses identified a shared mutational trunk of driver alterations across all tumor regions, reinforcing their clonal relationship [12, 27]. However, exclusive alterations emerged within the NEC (and also the adenocarcinoma) compartments, including CNVs (ovary), mutations (ileocecal), and increased copy numbers of shared CNVs (gastric)—a finding partially reflected in transcriptomic profiles. In the ileocecal MiNEN, while *RB1* and *PTEN* mutations were present throughout the NEC population, further sequencing revealed distinct alterations in morphologically defined subpopulations: a *CTNNB1* mutation in the large-cell NEC and additional CNVs in the small-cell NEC. In the gastric MiNEN, although the SC-NEC component lacked exclusive alterations, it displayed significantly higher *MYC* amplification levels compared to the glandular compartments, where this amplification was present at lower levels. This observation suggests that the degree of Myc activation—here driven by an increase in *MYC* copies—may have played a pivotal role in driving neuroendocrine differentiation, aligning with previous functional studies in gastric NEC [11].

To assess whether ST could reliably assign transcriptional profiles to histological regions, we examined transcriptomic clustering in all three cases. ST successfully delineated distinct transcriptomic profiles corresponding to annotated histopathological regions. This analysis revealed diverse gene expression trajectories along the progression from premalignant lesions to NEC, while also highlighting similarities within the NEC compartments of the investigated tumors. One prominent finding from gene set enrichment analysis (GSEA) was the consistent downregulation of immune-response-associated pathways (e.g., inflammatory response, interferon-γ/α response) in NEC components compared to adenocarcinoma regions. This reduction in immunogenicity may contribute to the aggressive behavior typically observed in NEC. Moreover, the NEC components across all cases displayed upregulation of proliferation-associated pathways (e.g., E2F, G2M*,* Myc activation, and Hedgehog signaling) alongside downregulation of apoptotic signaling. These findings are consistent with the highly proliferative nature of NECs and align with results from a recent spatial transcriptomics-based study on gastric MiNEN, which reported similar observations [19]. The approach used in our study, which aimed to identify general transcriptomic signatures of the tumor components rather than individual gene alterations to obtain an initial comparative overview, conceptually differs from that of another recent ST-based study on a single case of combined large-cell neuroendocrine carcinoma of the lung, where the analysis focused primarily on a single gene, *SMC1A* [14].

To investigate whether transcriptomic heterogeneity exists within histologically indistinguishable tumor areas, we performed a separate analysis of the ST profiles of the NEC components. Surprisingly, this revealed a variety of distinct subclusters, each defined by dominant transcriptional hallmarks, in both the ileocecal and ovarian NEC components. Correlating these divergent transcriptomic signatures with predicted responses to chemotherapies revealed differential response probabilities between the subclusters. Notably, this variability was observed for key drugs such as 5-FU, irinotecan, temozolomide, and paclitaxel. These exploratory findings are significant, as they suggest that vastly different gene expression profiles may coexist in morphologically similar tumor regions, potentially driving differential treatment responses and contributing to the limited chemotherapy response observed in many extrapulmonary NECs [25, 26, 28]. Interestingly, the SC-NEC component in the gastric mixed adenocarcinoma-NEC did not exhibit statistically significant transcriptomic clusters. This unexpected finding highlights that even the presence of transcriptomic heterogeneity itself is inconsistent across MiNEN cases, suggesting that heterogeneity is, in fact, a heterogeneous feature of MiNEN—further underscoring the biological complexity of these tumors.

While our study provides valuable insights into the transcriptomic and genomic landscape of MiNEN, specifically mixed adenocarcinoma-NECs, certain limitations must be acknowledged. The sample size was limited to three cases from different organs, which may restrict the generalizability of our findings. These cases were intentionally selected to represent heterogeneous histopathological patterns at diverse anatomical sites, aiming to comprehensively exemplify the spectrum of possible intratumoral heterogeneity in MiNEN. Notably, even with only three cases, the combined spatial transcriptomics and genomic data generated in this study offer remarkable depth, providing an extensive dataset that would exceed the scope of a single manuscript if all potentially analyzable facets of this dataset were fully explored within a single study. Therefore, to maintain focus, we limited our analysis to the tumor compartments and prioritized a deeper investigation of NEC subclusters, given their role as the biologically dominant component in MiNEN. Furthermore, while our findings suggest potential relevance for treatment response, they remain conceptual at this stage and warrant functional validation to determine the clinical significance of the identified transcriptomic subclusters. Lastly, although our approach uncovers a novel layer of heterogeneity that is linked to histomorphology in an unprecedented manner, other advanced technologies such as imaging mass spectrometry, in situ sequencing, or spatial epigenomics hold great promise to provide additional insights at different molecular levels.

In conclusion, our study highlights the remarkable molecular complexity of MiNEN, specifically mixed adenocarcinoma-NECs, revealing that their intratumoral heterogeneity extends beyond previously recognized boundaries. Through the combination of next-generation sequencing and spatial transcriptomics, we demonstrated that while mixed adenocarcinoma-NECs share a common clonal origin, distinct genetic alterations emerge within the NEC compartments, reflecting a degree of compartment-specific evolution. Importantly, spatial transcriptomics revealed substantial differences not only between the adenocarcinoma and NEC components but also within morphologically indistinguishable NEC regions themselves. This transcriptomic variability was accompanied by differential predicted responses to key chemotherapeutic agents, underscoring that distinct transcriptional subclusters may influence treatment response in NECs. Notably, the absence of transcriptomic subclusters in the SC-NEC of the gastric MiNEN further illustrates that even the presence of heterogeneity itself is inconsistent across MiNEN, reinforcing the complexity of these neoplasms. Together, these findings provide new insights into the diverse molecular landscape of MiNEN and highlight the potential impact of transcriptional heterogeneity on therapeutic resistance, underscoring the need for refined treatment strategies in these highly aggressive tumors.

## Supplementary Information

Below is the link to the electronic supplementary material.

ESM 1 (XLSX 24.2 KB)

## Data Availability

Data is provided within the manuscript or supplementary information files. Tissue and data from this manuscript are stored at the Institute of Pathology, Philipps University Marburg und University Hospital Marburg, Marburg, Germany and are available from the corresponding author upon reasonable request.

## Abbreviations

EMT: Epithelial-mesenchymal transition
LC-NEC: Large-cell neuroendocrine carcinoma
MANEC: Mixed adenoneuroendocrine carcinomas
MiNEN: Mixed neuroendocrine–nonneuroendocrine neoplasms
SC-NEC: Small-cell neuroendocrine carcinoma
STIC: Serous tubal intraepithelial carcinoma
